# Clinical effect of day case arthroscopic surgery in tibial-eminence fracture in adults using button plates

**DOI:** 10.3389/fsurg.2022.899438

**Published:** 2022-09-29

**Authors:** Xiaohui Xu, Huayi Wang, Fengguo Cui, Feng Guo

**Affiliations:** ^1^ Department of Orthopaedics, QiLu Hospital of ShanDong University, Dezhou Hospital, Dezhou, China; ^2^ Department of Orthopaedics, Xijing Hospital, Air Force Military Medical University, Xi’an, China; ^3^ Department of Orthopaedics, Beijing Rehabilitation Hospital, Capital University of Medical Sciences, Beijing, China

**Keywords:** arthroscopic fixation, fracture, tibial eminence, clinical efficacy, patient satisfaction, button plates (TightRope)

## Abstract

**Background:**

The tibial-eminence fracture (TEF) is an anterior cruciate-ligament avulsion fracture with a low incidence. Many surgical techniques have been described, but none of them allow early functional exercise, and there are many postoperative complications.

**Purposes:**

This study aimed to evaluate the early clinical efficacy and complications of day case arthroscopic-surgery treatment of adult TEF with button plates.

**Methods:**

We retrospectively analyzed patients with TEF treated with arthroscopic surgery. Clinical subjective evaluation included International Knee Documentation Committee (IKDC) subjective score, Lysholm Knee Score, and Visual Analog Scale (VAS) score. Knee joint scores were evaluated by Lysholm score. Clinical objective assessment included the Lachman test, anterior-drawer test (ADT), IKDC, and range of motion. We assessed patient quality of life using a life summary table. Assessment of fracture healing and internal fixation was based on lateral x-rays of the knee joint. We measured and evaluated patient satisfaction at the last follow-up in accordance with Marsh criteria.

**Results:**

At final follow-up (average follow-up time, 28.23 ± 3.14 months), we evaluated results from 22 patients (22 knees). Average patient age during surgery was 33.64 ± 6.96 years. Average time from injury to surgery was 6.59 ± 1.47 h. Postoperative function was better than pre-operative function in all patients. IKDC subjective score, Lysholm score, and VAS score were better at final follow-up than before surgery. Differences in Lachman test and ADT scores before and after surgery were statistically significant. According to Intra-articular button position classification, 6 patients (6 knees) showed ideal position (A), 16 patients (16 knees) showed nearly ideal position (B), and none of the patients had nonideal position (C). The fractures of 22 patients healed completely; 2 patients had a 5°–10° knee joint dysfunction, and 1 had an abnormal knee sound. According to intra-articular button position classification, the rate of ideal position was 100%. Patient satisfaction rate was 81.8%.

**Conclusion:**

Day surgery using double-button plates to treat TEF could achieve anatomical reduction, power and stability, as well as good clinical efficacy.

## Introduction

The tibial-eminence fracture (TEF) is an anterior cruciate-ligament (ACL) avulsion fracture with a low incidence. Previous studies have reported that TEF commonly occurs in children and adults (1). Currently, up to 40% of these fractures occur in adults (2). TEFs were first described by Meyers and McKeever in 1959. They are divided into four types: type 1, which is nonreducible; type 2; and types 3 and 4, which require surgical treatment (1, 3). The current treatment plan for displaced TEFs involves anatomical reduction of the fracture, reconstruction of the ACL, early functional exercise, restoration of knee joint function, and quality of life (QoL) improvement.

Fracture treatment options in adults include incision or arthroscopic screws, steel wires, metal sutures, or metal-free sutures (4). Regardless of fixation type, the purpose is to achieve stability, reconstruction, and early functional exercise. However, none of the above methods permit early functional exercise, and there are many postoperative complications. Therefore, developing a better surgical plan to facilitate patient recovery is crucial. In this study, we adopted a new fracture treatment plan based on double-button fixation to evaluate the clinical efficacy of day surgery.

## Materials and methods

This study was conducted in accordance with the Declaration of Helsinki and approved by the Institutional Review Board of our institution. Enrolled patients signed their informed consent (Approval No. 2020042).

We retrospectively analyzed the clinical efficacy and complications of double-button plate surgical treatment in TEF patients from April 2017 to April 2019. All patients were operated on by the same group of surgeons. The sole inclusion criterion was no obvious contusion of the skin over the knee joint. Exclusion criteria were as follows: (1) no closed epiphyses; (2) arteriovenous injury; (3) previous meniscal resection; (4) abnormal imaging findings; (5) lack of consent to participate in the study; (6) multiple-ligament injury; (7) ACL rupture; and (8) previous knee dislocation or old TEF.

All patients received clinical and radiological examinations before surgery, including computed-tomography (CT) and magnetic-resonance imaging examination.

### Clinical evaluation

Postoperatively, we followed up on patients in the outpatient department at 1 week, 4 weeks, 6 weeks, 3 months, 6 months, and annually thereafter. Clinical results were evaluated by Visual Analog Scale (VAS) score, Lysholm Knee Score, International Knee Documentation Committee (IKDC) subjective score, range of motion (ROM), Lachman test score, and ACL stretch test [anterior-drawer test (ADT)] score. Lysholm scores were graded as excellent (87–100), good (77–86), general (67–76), or poor (<67) (5). Lachman test scores were graded as 0 (no difference), 1 (1–5 mm laxity), 2 (5–10 mm laxity), or 3 (>10 mm laxity). We evaluated radiographs using anteroposterior and lateral x-rays. These outcome measures are regarded as essential for evaluating fracture healing and knee function in patients with TEF. Disappearance of the fracture line indicated healing.

### Quality of life

Patients’ QoL was evaluated using Short Form 12 (SF-12) profiles, including a physical-component summary (PCS) and a mental-component summary (MCS).

### Satisfaction

Patient satisfaction, evaluated at the last follow-up, was based on Marsh’s six-level classification (6): extremely satisfied, satisfied, partly satisfied, neither satisfied nor dissatisfied, somewhat dissatisfied, and very dissatisfied.

## Surgical technique

Depending on the patient’s condition, we performed the arthroscopic procedure under general or epidural anesthesia. The patient was placed in the supine position, and a lower-extremity tourniquet was used.

**Figure 1:**
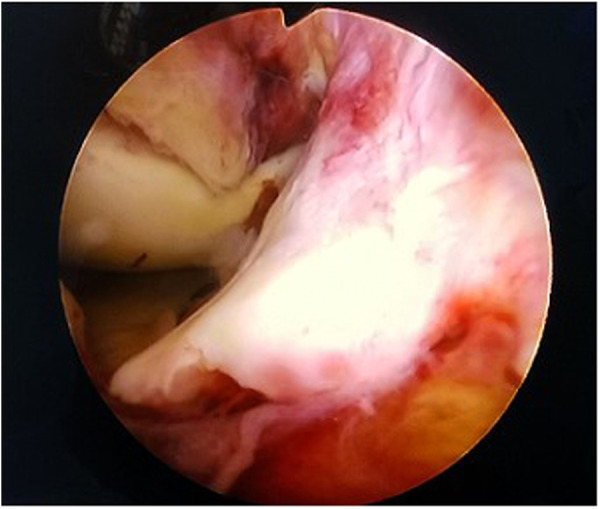
Arthroscopic view: type III fracture with ligament being obstacle to reduction.

The first step was using the arthroscopic anterolateral, anteromedial, and patellar approaches to explore the joint cavity. Arthroscopy was continued to flush the joint cavity. We used an electric scalpel and radiofrequency electrocautery to clean up the blood clots and clean the synovium of the fractured end. First, the fracture was cleaned; then, if it was type 4, we tied it with a non-absorbable thread to modify it into a type 3 fracture. The second step was to carefully explore the surroundings and use the rear-drawer test to facilitate reduction. After resetting the bone block, we used a 1.0-mm Kirschner wire for temporary fixation (Figure 1).

**Figure 2:**
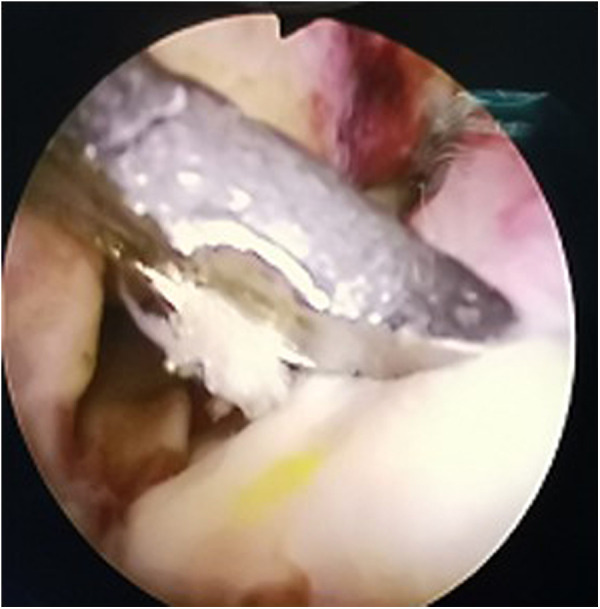
Arthroscopic view: reduction of fracture block with reducer.

**Figure 3:**
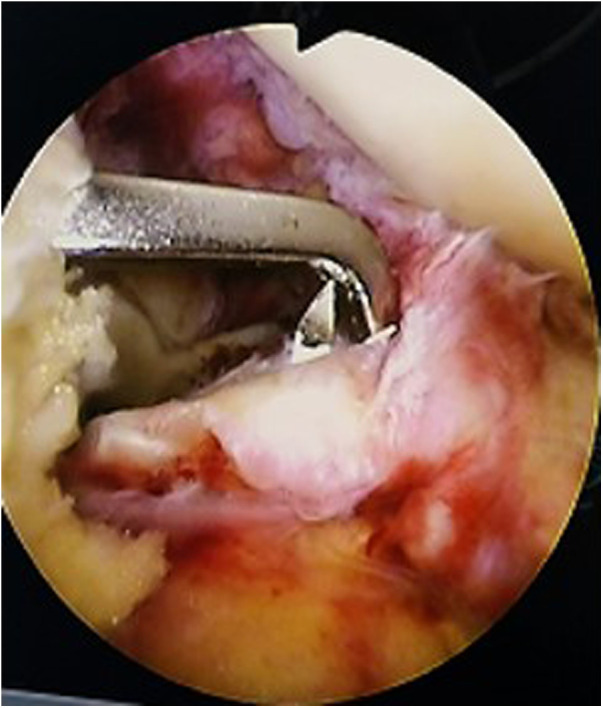
Arthroscopic view: the guide hole the reduction and determines the pain placement, insert the guide needle into the guide to set the position.

**Figure 4:**
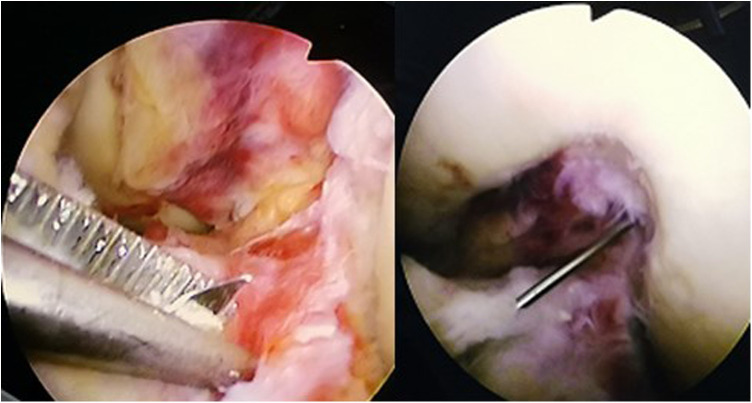
Arthroscopic view: maintain the position of the guide needle and insert the hollow drill.

**Figure 5:**
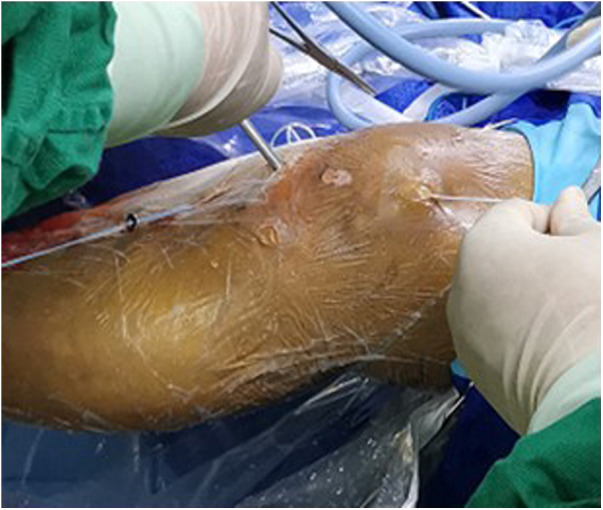
The assistant pulls the white cord in order to pull the button plate out through the hole.

**Figure 6:**
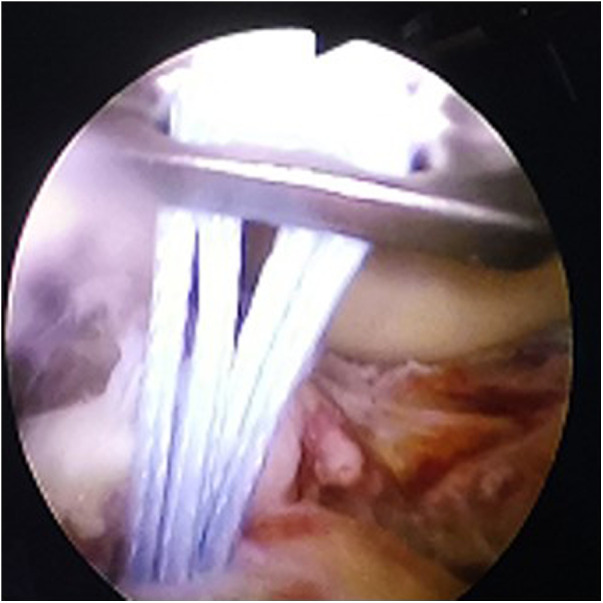
Arthroscopic view: pull and flip button plate.

A C-type (point-to-point) guide positioner was placed on the tibial intercondylar eminence to maintain the reset simultaneously into the guide pin. (We suggest that the intra-articular plate be placed in the first half to first third of the free bone to prevent “seesawing.”) In the third step, we introduced a 2.4-mm threaded needle through the guide, crossing the tibial cortex and tibial eminence, and terminating at the ACL insertion. A 4.5-mm tunnel was drilled along this threaded needle, which allowed the surgeon to insert the oblong button down through the osseous tunnels. A guidewire was successively passed through the cannulated drill, which we used to prepare passage for an intra-articular button (TightRope; Arthrex, Inc., Naples, FL, USA) (Figures 2–4). The button was turned and placed over the tibial eminence under arthroscopic guidance. Then, we tightened the traction sutures. These sutures were tied on the round extra-articular metal button, which was created to keep the fracture fragment reduced (Figures 5, 6).

## Postoperative treatment plan

Knee joints were treated postoperatively with rehabilitation program. Patients wore a knee brace for adjustable knee flexion and extension for the first day. Full-weight–bearing exercise was prescribed at 0°–30° for the remainder of the first week, 0°–50° for the second week, 0°–60° for the third week, 0°–75° for the fourth and fifth weeks, and 0°–90° for the sixth week. Six weeks after surgery, the brace was removed, and knee flexion and extension were strengthened *via* unrestricted functional exercise.

## Statistical analysis

All data were analyzed using SPSS version 18.0 (IBM Corp., Armonk, NY, USA). We calculated means and standard deviations for IKDC, VAS, SF-12, and Lysholm scores. Ratios were calculated for categorical variables (Lachman grade, ADT, and overall IKDC grade) and compared using the *χ*^2^ test. We used a paired *t*-test to compare Constant score before surgery with that at the last follow-up. The level of significance was set at *P* < 0.05.

## Results

We included a total of 32 patients (32 knees) with TEF types 2–4 according to Meyers and McKeever classification. Ten patients (10 knees) were lost to follow-up; a total of 22 patients (22 knees) with displaced TEFs who had undergone arthroscopic treatment between April 2017 and April 2019 completed the study at the last follow-up.

Average follow-up duration was 28.23 ± 3.14 months (range, 25–36 months). This study included 12 men and 10 women, with a mean age of 33.64 ± 6.96 years. Mean time interval between injury and surgery was 6.59 ± 1.47 h. Twenty patients (90.91%) had Meyers and McKeever type 3 avulsion fractures, and two (9.1%) had type 4 fractures; 20 out of 22 patients had concomitant meniscal and cartilage injuries (7), and seven had simple fractures. At the last follow-up, two patients had 5° and 10° loss of normal knee joint function compared with the normal contralateral knee joint.

All patients restored the ROM of the involved knee to a completely normal range or a range with an acceptable deficit of <10° compared with that of the normal contralateral knee. One patient had sporadic abnormal sound in the knee, but function was not affected.

## Subjective function assessment

### VAS score

VAS score declined significantly from 7.00 ± 1.35 before surgery to 1.55 ± 0.86 at the last follow-up (*t* = 25.31, *P* < 0.001; Table 1).

**Table 1:** Scores differences between perioperatively and postoperatively.

	VAS	IKDC Subjective	Lysholm Score	SF-12 PCS	SF-12 MCS
Perioperatively	7.00 ± 1.35	37.36 ± 4.75	6.41 ± 4.32	32.47 ± 3.71	43.69 ± 2.96
Postoperatively	1.55 ± 0.86	90.09 ± 2.27	96.41 ± 0.59	41.61 ± 8.36	54.60 ± 2.75
*t*	25.31	47.02	96.86	−4.27	−16.54
* P*	***	***	***	***	***

Values are reported as Means ± SD.

*** Means *P* < 0.001.

### IKDC subjective score

Mean IKDC score improved from 37.36 ± 4.75 perioperatively to 90.09 ± 2.27 postoperatively (*t* = 47.02, *P* < 0.001; Table 1).

### Lysholm knee score

Lysholm score increased from 6.41 ± 4.32 points before surgery to 96.41 ± 0.59 points at the last follow-up (*P* < 0.05), and this difference was statistically significant (*t* = 96.86, *P* < 0.001). According to Lysholm knee score, the excellent or good knee joint score had a rate of 100%.

## Quality of life and patient satisfaction

Mean PCS score increased from 32.47 ± 3.71 before surgery to 41.61 ± 8.36 at the last follow-up (*t* = −4.27, *P* < 0.001), while mean MCS score increased from 43.69 ± 2.96 before surgery to 54.60 ± 2.75 at final follow-up (*t* = −16.54, *P* < 0.001; Table 1).

Patient satisfaction was measured as suggested by Marsh (6). Six (27.3%) patients felt extremely satisfied, 12 (54.5%) felt very satisfied, 3 (13.6%) felt somewhat satisfied, and 1 (4.5%) felt neither satisfied nor dissatisfied. The satisfaction rate was 81.8%.

## Objective function assessment

### Anterior-drawer test

Out of 20 patients (20 knees) with positive perioperative ADT scores, only 3 knees showed positive ADT at the last follow-up (difference between before and after: *χ*^2^ = 26.33, *P* < 0.0001; Table 2).

**Table 2:** Patients with objective results of differences between perioperatively and postoperatively (ADT and Lachman test).

	ADT		Lachman test	
	(+)	(−)	(+)	(−)
Perioperatively	20	3	18	1
Postoperatively	2	19	4	21
*χ*^2^	26.33		26.77	
* P*	<0.0001		<0.0001	

Values are reported as the number of patients (%).

### Lachman test

Out of 18 patients (18 knees) with preoperative positive Lachman test scores, only one knee had a positive Lachman score at final follow-up, and the difference between before and after surgery was statistically significant (*χ*^2^ = 26.77, *P* < 0.0001; Table 2).

### Range of motion

The ROM of patients with serious functional limitations to knee extension/flexion increased from 11.77 ± 6.10 to 130.45° ± 9.55°, and no patients needed arthroscopic-release therapy. Knee movement returned to an acceptable normal range in 20 patients. One patient had a deformity of approximately 10° at the last follow-up.

## Radiographic results

According to our evaluation of lateral–knee joint x-ray results, all patients achieved anatomical reduction of the bone block, and the fracture block healed within 3 months after the operation.

## Intra-articular button position

**Figure 7:**
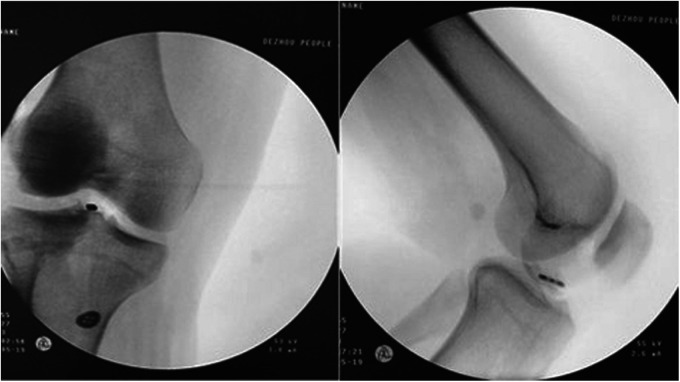
Postoperative anterior-posterior and lateral view radiographs. The ideal position (**A**).

**Figure 8:**
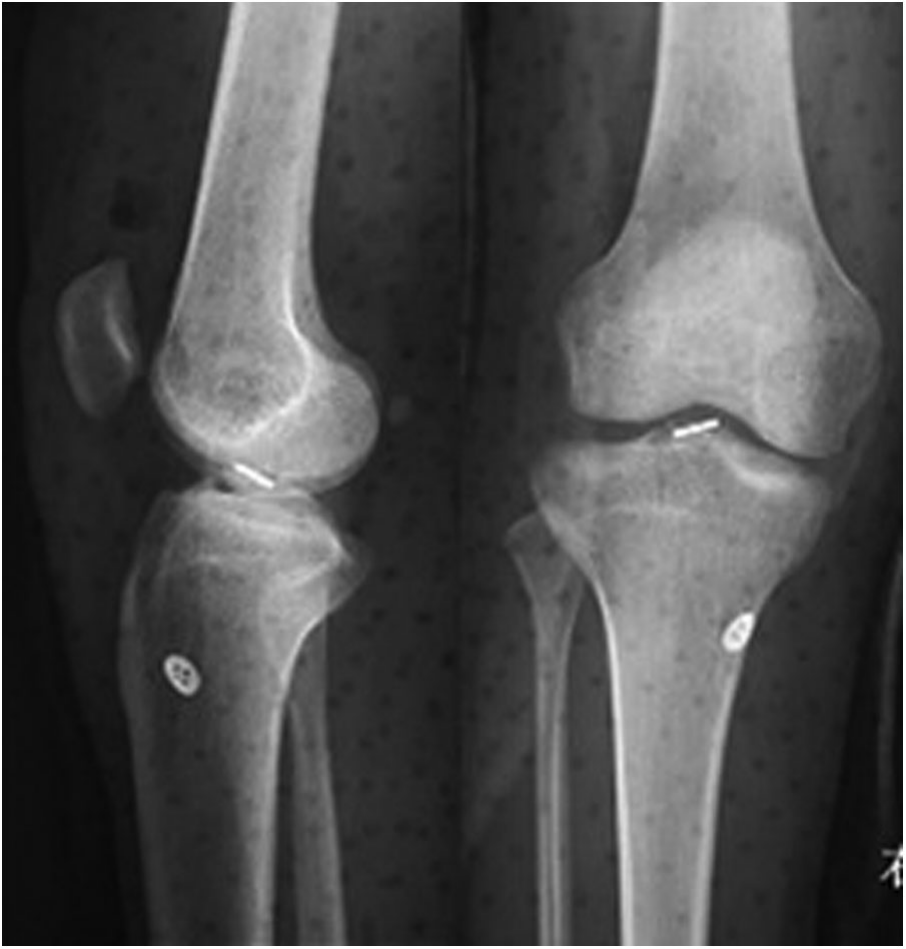
Postoperative anterior-posterior and lateral view radiographs. The nearly ideal position (**B**).

Lateral–knee joint x-rays indicated that the relationship between the long axis of the rectangular button loop in the joint and the sagittal plane of the human body could be categorized into three states: ideal, nearly ideal, and nonideal. The ideal position (A) was the long axis of the steel plate being parallel to the sagittal plane of the human body (Figure 7). The nearly ideal position (B) implied that an angle existed between the long axis of the plate and the sagittal plane of the body (Figure 8). The nonideal position (C) was the plate’s long axis being perpendicular to the body’s sagittal plane. According to this classification, 6 patients (6 knees) showed ideal position (A), 16 patients (16 knees) showed nearly ideal position (B), and none of the patients had nonideal position (C).

## Complications

Two patients showed a loss of 5° and 10° knee joint motion compared with the normal contralateral knee joint at the last follow-up. One patient (1 knee) had twisting pronunciation or abnormal sound, without alteration of knee function. There was no infection in any of the 22 patients.

## Discussion

Most adult TEFs are caused by trauma, especially the high-energy traumata of car accidents, falls, and certain other injuries (2, 8); these fractures are often accompanied by ligament and/or meniscal damage. The treatment plan for these patients is to provide elastic quality, tough stitching, and rigid hard-metal, fork-fixed avulsion reconstruction of the bone and ligament. All treatment regimens attempt to rebuild ACL tension and ligament proprioceptive function.

The tensile force of the native ACL (9, 10) during normal human activities is 500 N. The mean force of TEF is about 2500 N (10, 11). Based on the biomechanical properties of two metal buttons (TightRope; Arthrex, Inc., Naples, FL, USA), the mean vertical force in static load leading to failure is 982 N, and the mean anterior force in static load leading to failure is 627 N (12). The ultimate tensile force of this button system is strong enough to fix the fracture and restore the ACL (13, 14). It also illustrates the biomechanical properties and thus the feasibility of the button plates that can be used to treat TEF.

The treatment plan includes conservative management for type 1 nondisplaced TEFs. Surgical treatment is required for type 2 TEFs if the reduction is not anatomical (7, 15) and for all type 3 and 4 fractures (16, 17). Successful arthroscopic reduction and fixation have been described in recent studies (14).

With the use of arthroscopy in treatment, early activity and rapid recovery can be achieved, and hospital stay can be shortened. Treatment options reported so far include purse nails, cancellous bone screws, Kirschner wires, U-shaped nails, threaded rivets, sutures, and wire fixation. However, previous studies have reported that using suture fixation technology can help achieve good results.

Suture and rivet technology can achieve fixation of tibial intercondylar-ridge fractures and reconstruction of anterior-fork ligament tension (7, 18, 19). It has been reported that suture and screw fixation techniques are very effective in fixing fractures and reconstructing anterior-fork ligaments (7, 18, 19). However, the strength of these tools is not sufficient to favor the healing of fractures; most of them require a fixed full-knee extension position and non–weight-bearing exercise for a long time, which leads to knee joint adhesion and low activity. After treatment with these technologies, patients have low QoL and poor satisfaction.

**Figure 9:**
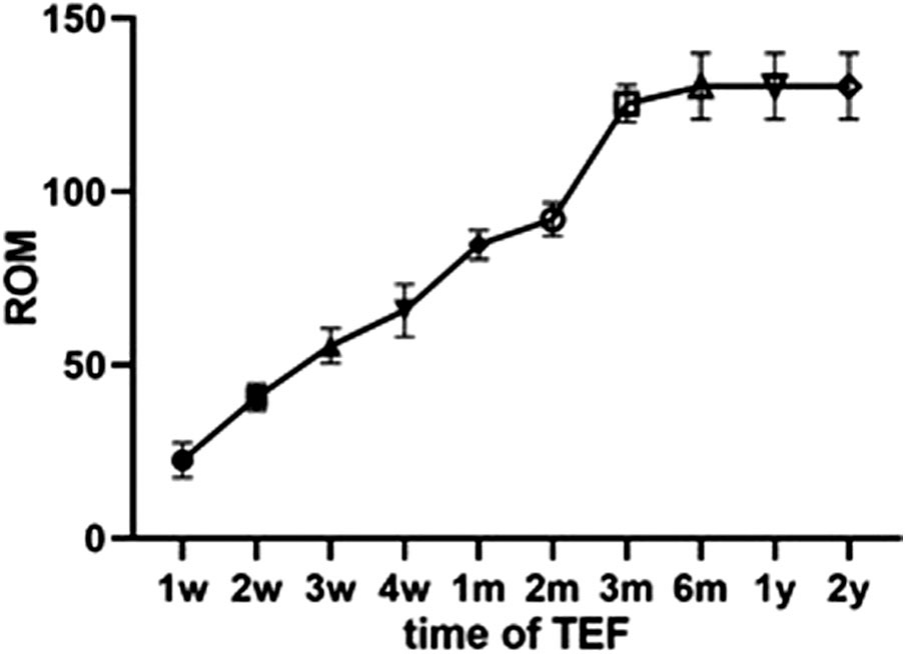
Changes of range of motion of knee joint after TEF operation.

The Chinese Ambulatory Surgery Alliance defines “day surgery” as a planned surgery other than outpatient surgery, 24 h after which the patient is discharged. We performed the arthroscopic double-button fixation technique on day cases, achieving good function and relatively excellent knee joint scores; this technique yielded the same or better results than other approaches (5, 13, 20, 21). There have been reports of this treatment plan in the literature, but few evaluation indicators are included (9, 22). In this day surgery study, patients received a double-button plate, which has both rigid and elastic characteristics. They were instructed to perform early functional exercises and put their full weight on the affected leg starting on day 2 after surgery. In contrast to the available literature, arthrofibrosis can be effectively avoided by continuously increasing the range of activities (20), and we suggest such an aggressive regimen to secure the fixation (Figure 9). At the last follow-up, average knee mobility was about 130.45° (range, 118–145°), which was comparable to or better than previously reported results (13, 20, 21). We believe that early day case arthroscopic surgery of TEF achieves better immediate surgical effect with more-favorable cost effectiveness.

At the same time, the SF-12 scores (PCS, MCS) of our patients increased significantly. This treatment allowed them to perform knee joint functional-rehabilitation exercises early, which can effectively reduce postoperative adhesion and stiffness caused by braking and increase the confidence of postoperative knee joint rehabilitation.

We believe that TEF patients often have ACL injuries, such as traction, which can affect the stability of the knee joint after surgery (2). In previous studies, in TEF carinal fractures, ACL injury was caused by traction during the fracture, causing >50% of the injury. However, this injury does not cause ligament rupture (2, 3), and there is no injury that could make the knee unstable.

Nonetheless, previous studies have reported that 44% of TEF patients with screw and wire fixation had physical and knee instability, requiring re-reconstruction of the ACL after this type of fracture. The re-reconstruction rate of the ACL in adults is reported to range from 7% (5 years after surgery) to 12% (15 years after surgery) (5, 6, 8). None of our patients needed ACL reconstruction. The injury composition was different from that in the previous report (5, 6, 8), which might have influenced the results at the last follow-up. Before performing the fixation, we thoroughly inspected the joint to exclude ligament rupture by arthroscopy. The satisfaction rate, which we measured as suggested by Marsh (6), was 81.8%.

Knee flexion and extension activities of all patients were severely restricted before surgery. Imaging examinations of all patients after 3 months showed that anatomical reduction of the bone block and fracture healing were achieved. At the last follow-up, ADT score was positive in three patients (3 knees, 13.63%), and Lachman score was positive in one (1 knee, 4.54%); our overall results were better than those of a previous study on screw or suture fixation (4). We considered that the reason for the positive ADT and Lachman scores might be postoperative anterior-fork ligament relaxation; in 10 of the 22 patients, there was also meniscal-ligament compression and cartilage injury. The meniscal-injury rate in our study was consistent with that reported in the literature; our patients were less involved in sports involving vigorous knee joint exercise such as football, basketball, or long-distance running. During follow-up, none of the patients had obvious discomfort, and none underwent secondary knee arthroscopy.

It is well known that no matter whether incision or arthroscopic surgery is performed for tibial intercondylar-ridge fractures, complications such as adhesion, fracture nonunion, dysfunction, loss, and relaxation occur (3, 13, 15, 20). Early rehabilitation exercises after fracture surgery can effectively restore knee function but can also increase the risks of refracture, non-union fracture displacement, increased bleeding, increased inflammatory response, and repeated knee swelling (13, 20). The postoperative recovery process for our patients was different from that described in previous similar reports (9, 22). In this study, we included more evaluation indicators, and we asked patients to bear their full weight on the affected leg on day 2 after surgery. Functional exercise was within the adjustment range of the brace. We believe that early functional exercise is conducive to knee rehabilitation and improves knee mobility (i.e., ROM). Steel-button plate fixation offers elastic fixation and promotes fracture healing (10, 11). Postoperative complications with this treatment protocol are significantly less common than have been previously reported in similar studies (7, 12–16). In this study, two patients had knee joint extension loss at the last follow-up, which was similar to findings in the existing literature; there was no joint release or joint ROM release under anesthesia. After discharge from the hospital, patients were urged to perform strengthening functional exercises of the knee joint at home in a timely manner, which could significantly improve the restricted movement of the joint. The proportion of patients with car accident trauma in this study was high, and there were often soft-tissue injuries around the knee joint. These injuries led to easy adhesion, causing knee joint dysfunction. Patients undergoing day case arthroscopic surgery do not need to wait long before the operation, and they can exercise earlier afterward.

**Figure 10:**
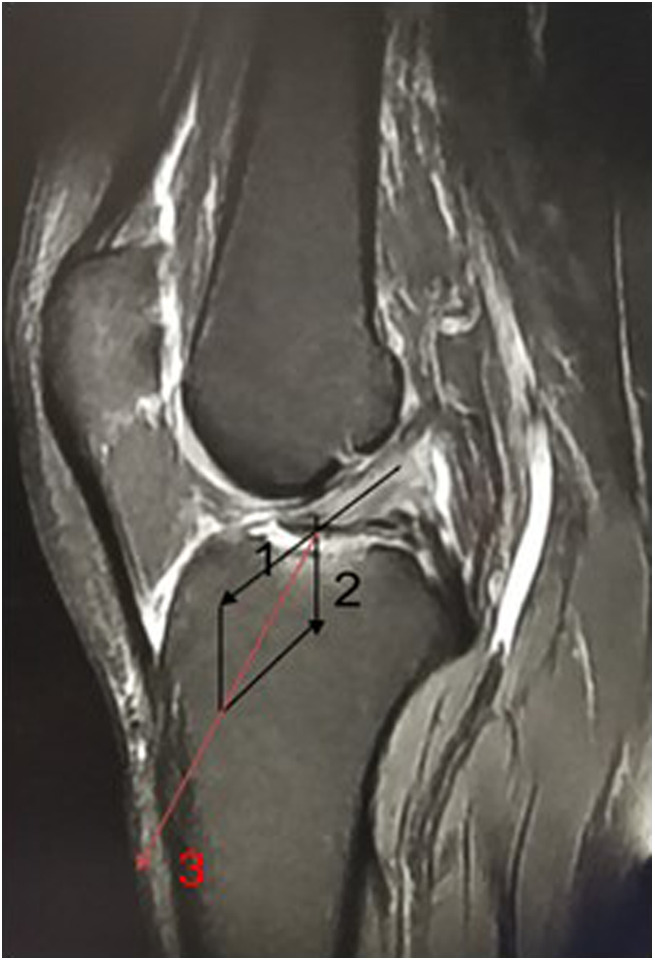
(1) The direction of the force line represents the tension direction of the anterior bifurcate ligament. (2) The direction of the force line represents the pressure direction of the fracture block. (3) The direction of the button where was fixed at the combind force direction, which was between the bone mass and the ACL.

The direction of the tunnel and the placement of intra-articular buttons can affect fracture healing and knee functional rehabilitation. The button plate requires anatomical reduction and fixation of the bone block and the combined-force direction of the ACL for the nail path during treatment, which can achieve maximum mechanical fixation. We suggest that the intra-articular plate be placed in the first half to first third of the free bone to prevent “seesawing.” If the plate is placed too far forward, such as at one third of the free bone, “excessive reduction” will occur in the front of the block and tilt will occur in the back, resulting in poor reduction. Moreover, the internal and external diameters of the free-bone fragments are larger when the intra-articular plate is placed backward, which is more advantageous to preventing the fragments from breaking during drilling. In addition, rotation of the plate can be prevented by placing it in the ACL. The nail path of our patients’ bone block was designed to follow the direction of force (Figure 10).

Femoral intercondylar presence is different in men and women; differences in femoral intercondylar width have been previously reported in the literature, with an average femoral intercondylar-terminus width of 14.5–24 mm (17, 20). However, the width of the intercondylar fossa in patients with osteoarthritis is narrower. The length of the long axis of the intra-articular loop plate is about 10 mm. We recommend that this axis be parallel to the sagittal plane of the knee joint, which can effectively avoid the impact of the button plate on the narrower intercondylar fossa and reduce damage. We routinely sutured and reinforced the intra-articular button into the ACL. However, during postoperative follow-up, we found that the button had rotated. This resulted in a risk of collision between the button plate and the intercondylar fossa. However, according to Intra-articular button position classification, 16 patient follow-ups were found in the continuous presence of loop rotation button, intra-articular rectangular loop into a fixed-position B type. 0 patient developed a C-type and had no knee discomfort during follow-up. During follow-up, 1 patient had postoperative bouncing weakness and abnormal noise when the knee joint moved, but knee flexion and extension function was good.

We do not recommend secondary surgery to remove the internal-fixation device because it is covered by soft tissue and ligament fibers after fracture healing. It is difficult to find and remove under arthroscopy. Secondary surgery increases costs and pain; however, if the intra-articular button body becomes loose in the knee, it must be removed.

## Conclusions

Day surgery for TEF using a double-button plate could significantly reduce hospital stay and preoperative waiting time. It could also accelerate rehabilitation of knee joint function, reduce rehabilitation time, and significantly improve patients’ early postoperative exercise capability.

## Data Availability

The original contributions presented in the study are included in the article/Supplementary Material, further inquiries can be directed to the corresponding author/s.
